# Supplementary material for: Solid-state polymer electrolytes for high-performance lithium metal batteries
Source: Nat Commun. 2019 Sep 27;10:4398. doi: 10.1038/s41467-019-12423-y (PMC6765010; doi:10.1038/s41467-019-12423-y)
Supplement: Supplementary file 1 — Supplementary Information [file 41467_2019_12423_MOESM1_ESM.pdf]

Supplementary Information

Solid-state Polymer Electrolytes for High-performance Lithium Metal Batteries

Snehashis Choudhury et al.

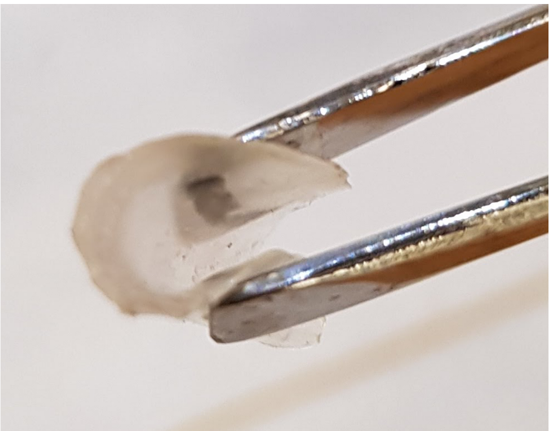

Supplementary Fig. 1: Photograph of the flexible crosslinked membrane.

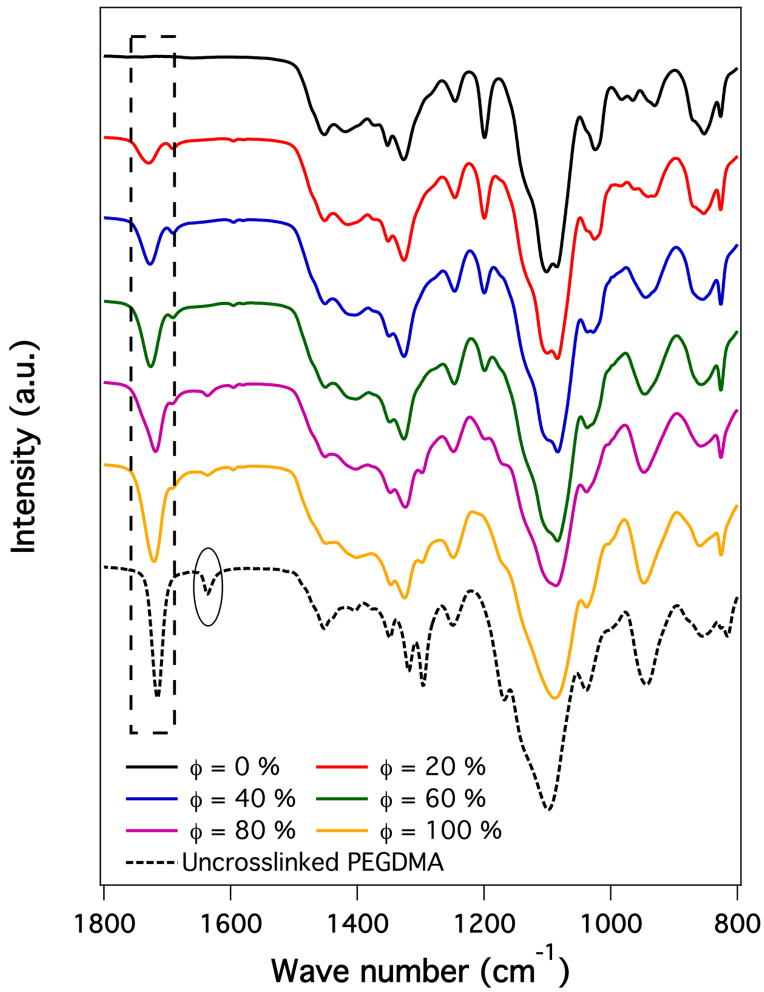

Supplementary Fig. 2: FTIR analysis of the crosslinked membrane at various PEGDMA content that shows C=O bond (at 1,700 cm^{-1}) shifting to lower intensity values as the volume percent PEGDMA is increased in solution. The circle marks the C=C bond (at 1650 cm^{-1}) that is only presented in the unreacted methacrylate groups.

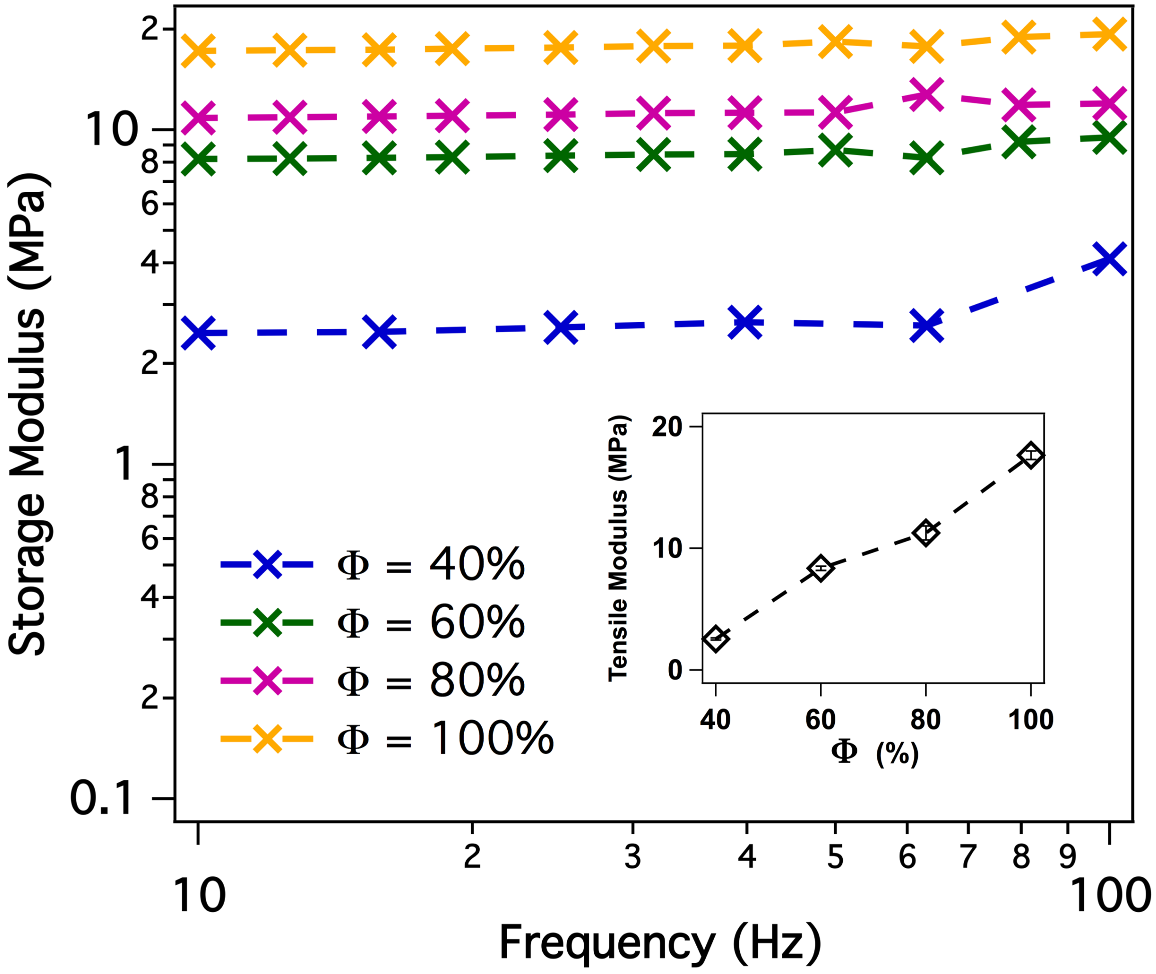

Supplementary Fig. 3: Tensile analysis of the crosslinked membrane at various PEGDMA content. The oscillatory tensile measurement was done on thin films at a low strain rate of 0.1% In the frequency range of 100-10Hz. The plateau modulus is plotted as a function of PEGDMA content in the inset.

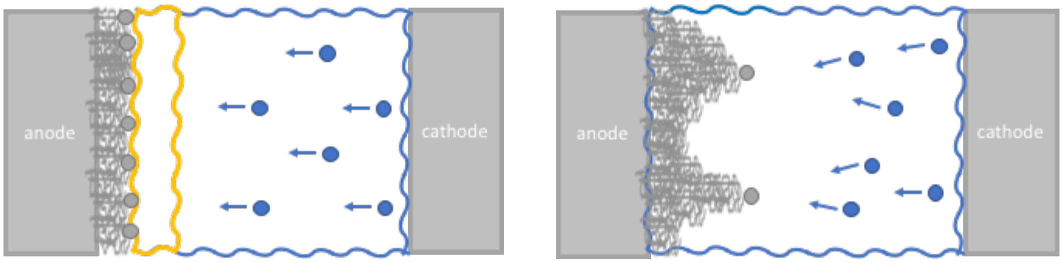

Supplementary Fig. 4: Schematic demonstrating the concept of stabilization using a solid polymer interphase.

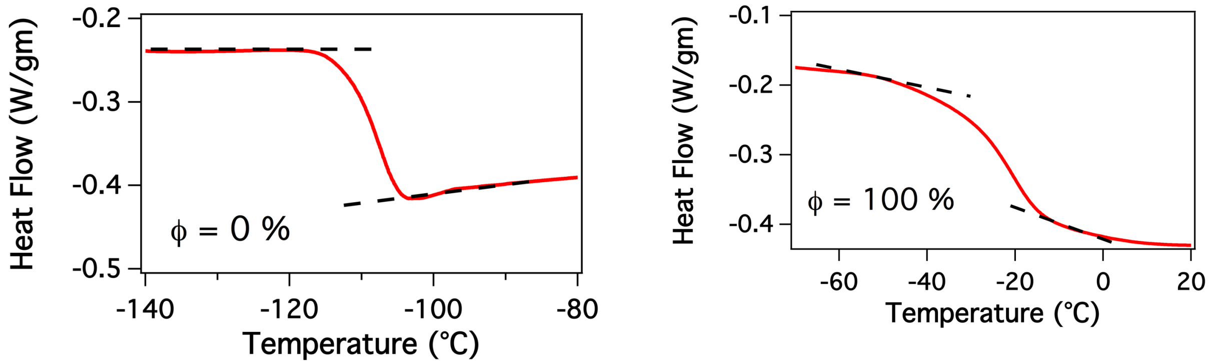

Supplementary Fig. 5: Thermograms obtained from Differential Scanning Calorimetry for pure diglyme (Φ = 0 %) and pure PEGDMA network (Φ = 100 %). The dotted lines mark the step-change in the heat-flow

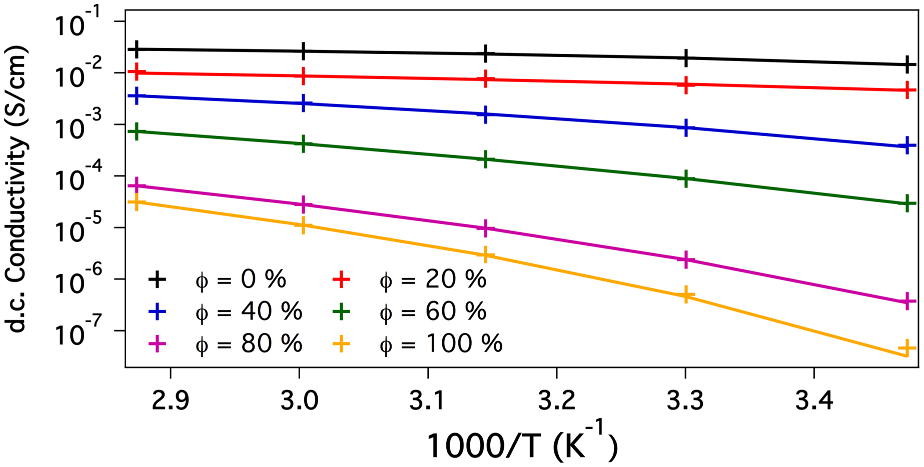

Supplementary Fig. 6: d.c. conductivity as a function of inverse absolute temperature. Measured values are shown as markers, and the data is fitted to Vogel Tamman Fulcher function.

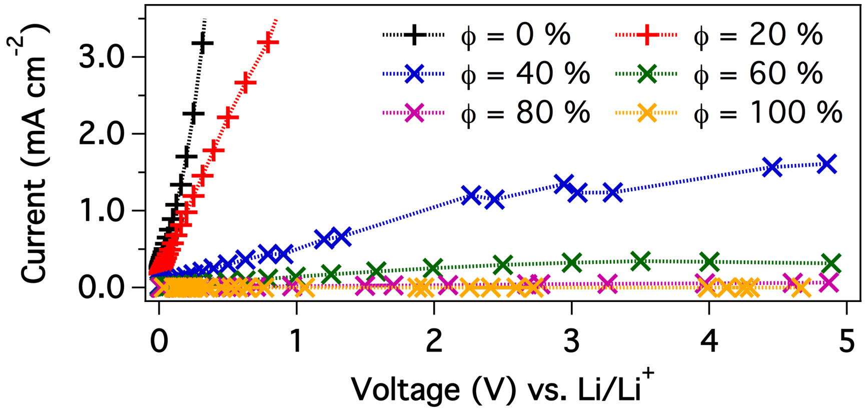

Supplementary Fig. 7: Current as a function of voltage. Divergence in current was seen for Φ=0% and Φ=20%, signaling the presence of electroconvection. For Φ=40% and beyond, the current reached a limiting value at higher voltages

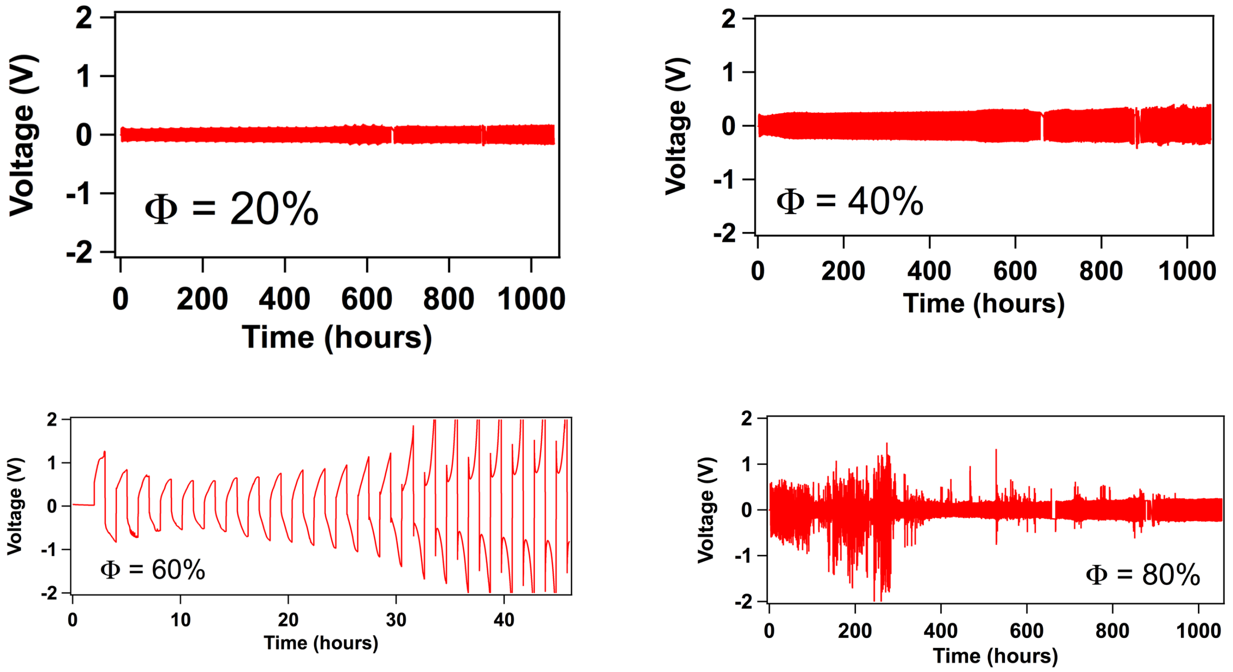

Supplementary Fig. 8: Voltage profiles of symmetric lithium cells with the solid polymer coatings on the electrode surface using the electrolyte 1M EC/DMC $LiPF_6$ with a celgard separator. The batteries were subjected to a constant current density of 0.5mA/cm^2 with each charge and discharge cycle being one hour long. The different samples represent the polymer coating comprising of varying fraction of PEGDMA content.

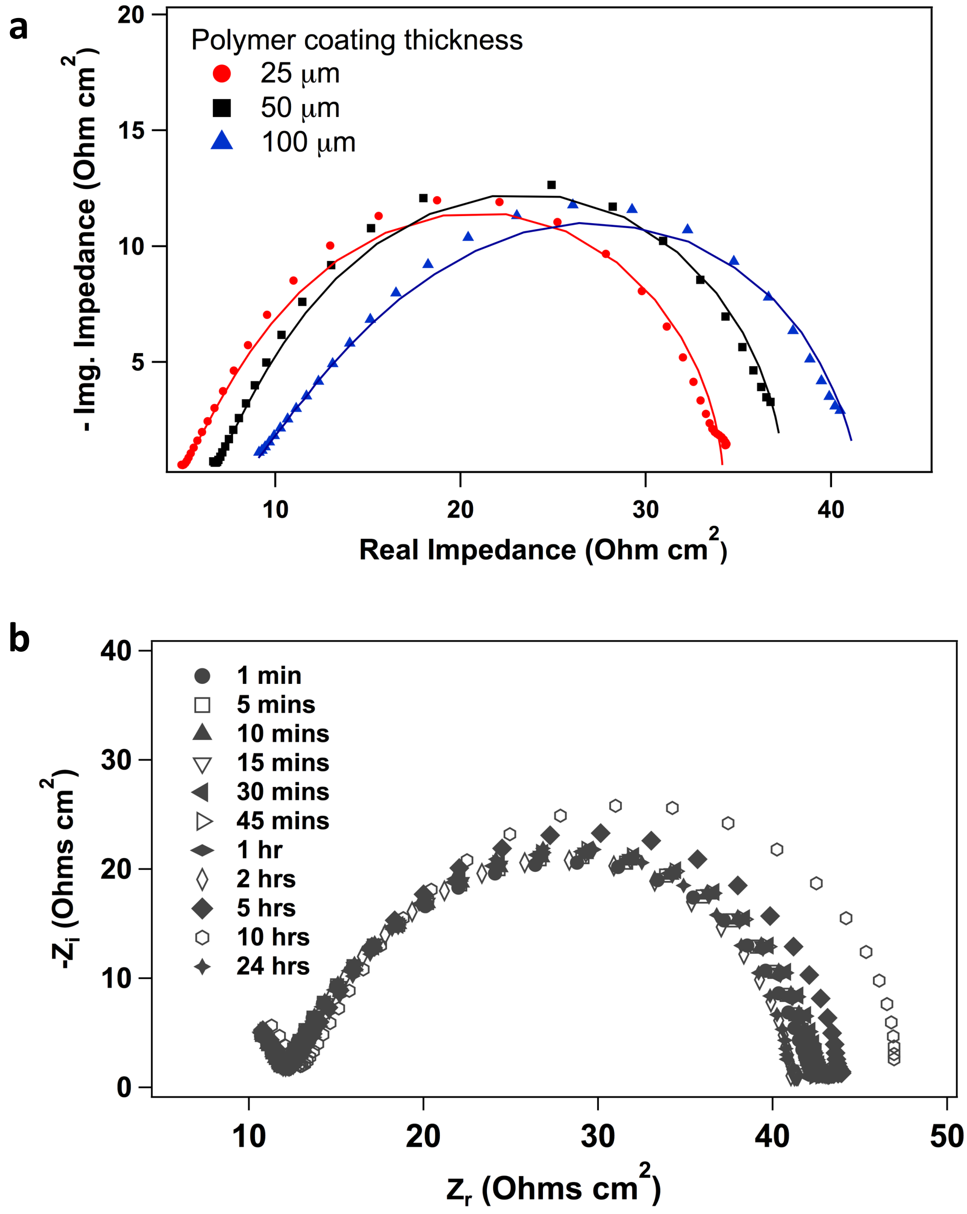

Supplementary Fig. 9: Impedance analysis of solid polymer interphase: (a) Nyquist plot showing the impedance spectra of symmetric Li cells coated with the solid polymer interphase, and the electrolyte utilized here is 1M EC/DMC $LiPF_6$. The measurement was for different thicknesses **(b)** Nyquist diagrams for the 100μm SPI coated symmetric lithium cells, where the measurements were performed at different times of aging

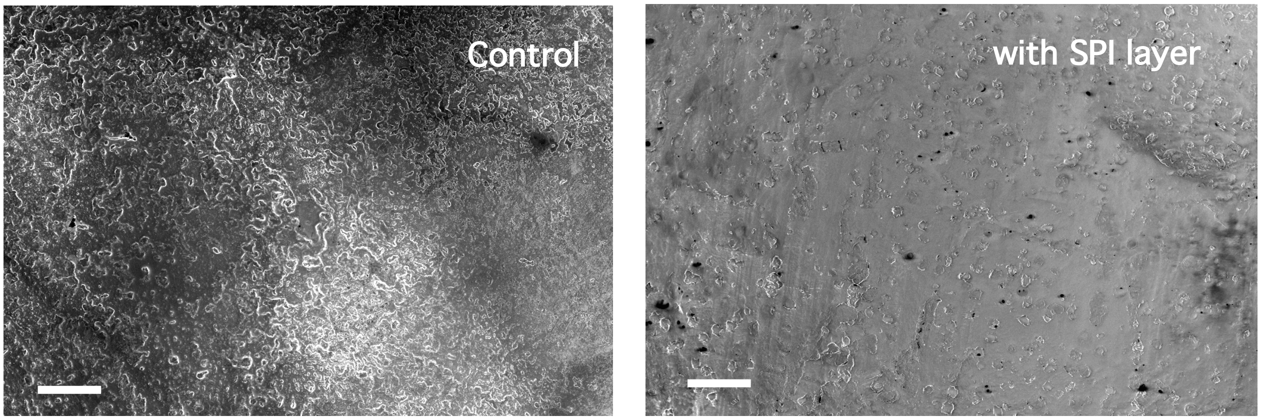

Supplementary Fig. 10: SEM micrographs showing the electrodeposition on metallic anode for one hour at the rate of 1mA/cm2. The left image is for a bare electrode while the right is for the electrode with a layer of the solid polymer coating. In both cases the electrolyte utilized was 1M $LiPF_6$ in EC/DMC. Both scale bars are 100μm.

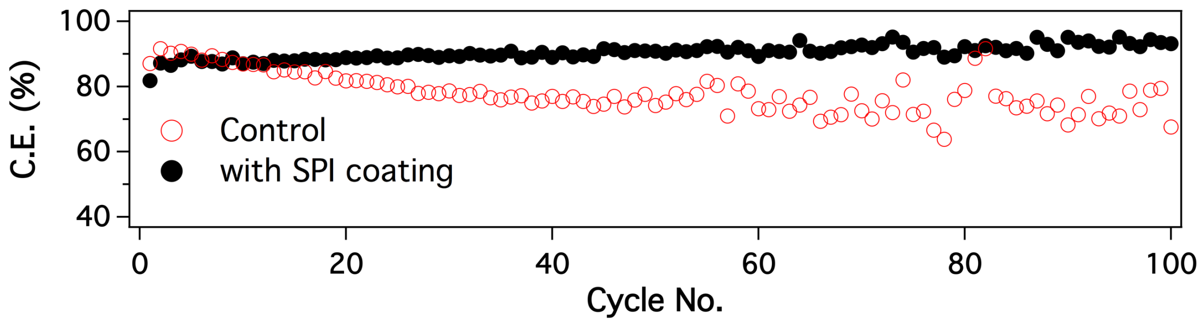

Supplementary Fig. 11: Coulombic efficiency measurement in Li || stainless steel coin cell with and without the solid polymer interphase at a current density of 1 mA cm^{-2} and capacity of 1 mAh cm^{-2}, using the 1 M $LiPF_6$ in EC/DMC electrolyte.

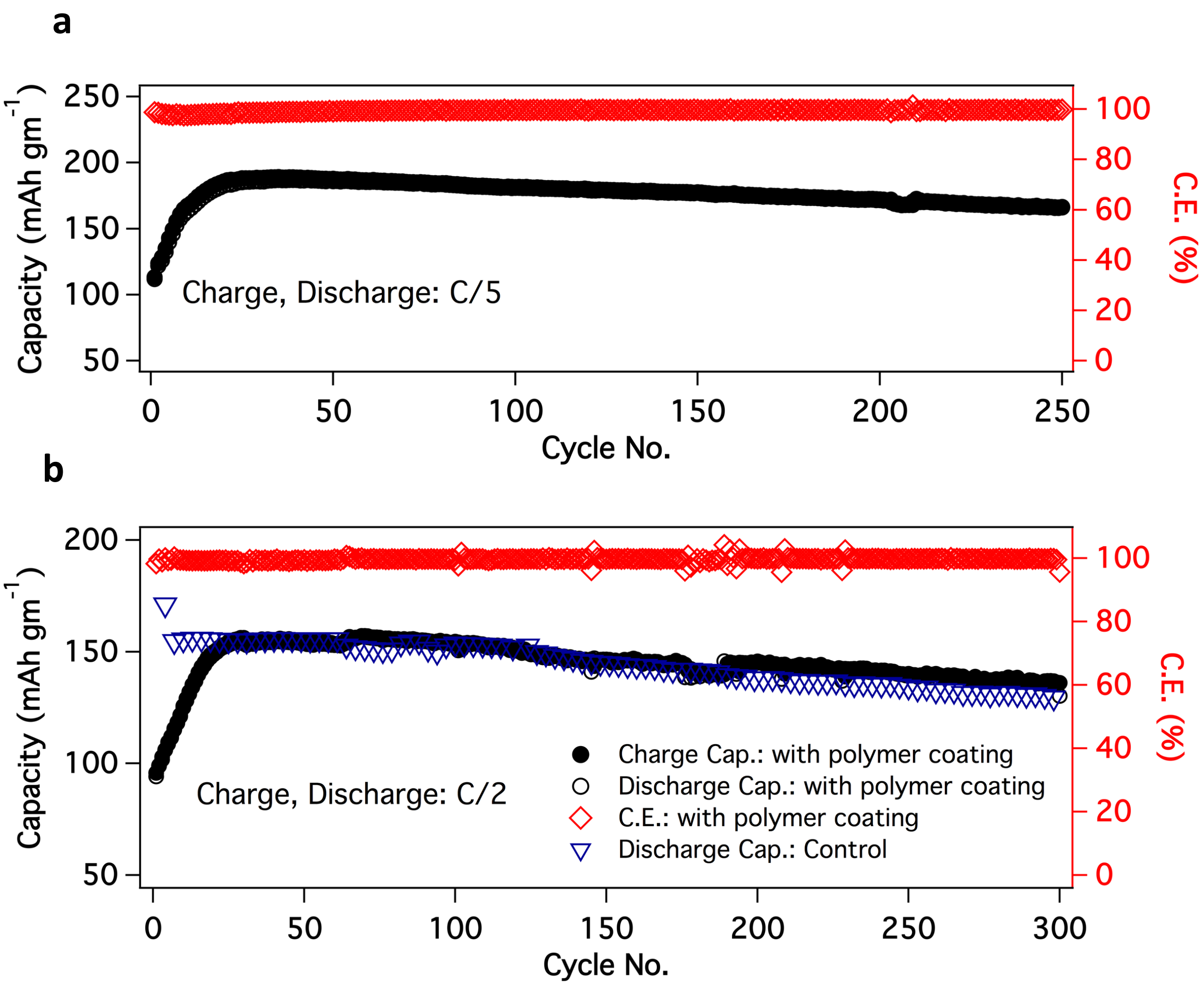

Supplementary Fig. 12: Full cell analysis of solid polymer interphases: Cycling performances for Li||NMC cells operated at a C-rate of (a) C/5 and (b) C/2. The data with black and red markers represents batteries having lithium metal electrode coated with the solid polymer interphase that comprises of the polymer network and diglyme, with PEGDMA content of 40%. The thickness of the polymer coating was ~100μm. The capacity of cathode is $2mAh/cm^2$ and the electrolyte used here is 0.6M LiTFSI, 0.4M LiBOB, 0.05 $LiPF_6$ in EC/DMC (1:1 by wt.). The blue marker in part b, represent the cells without the polymer coating using the same electrolyte.

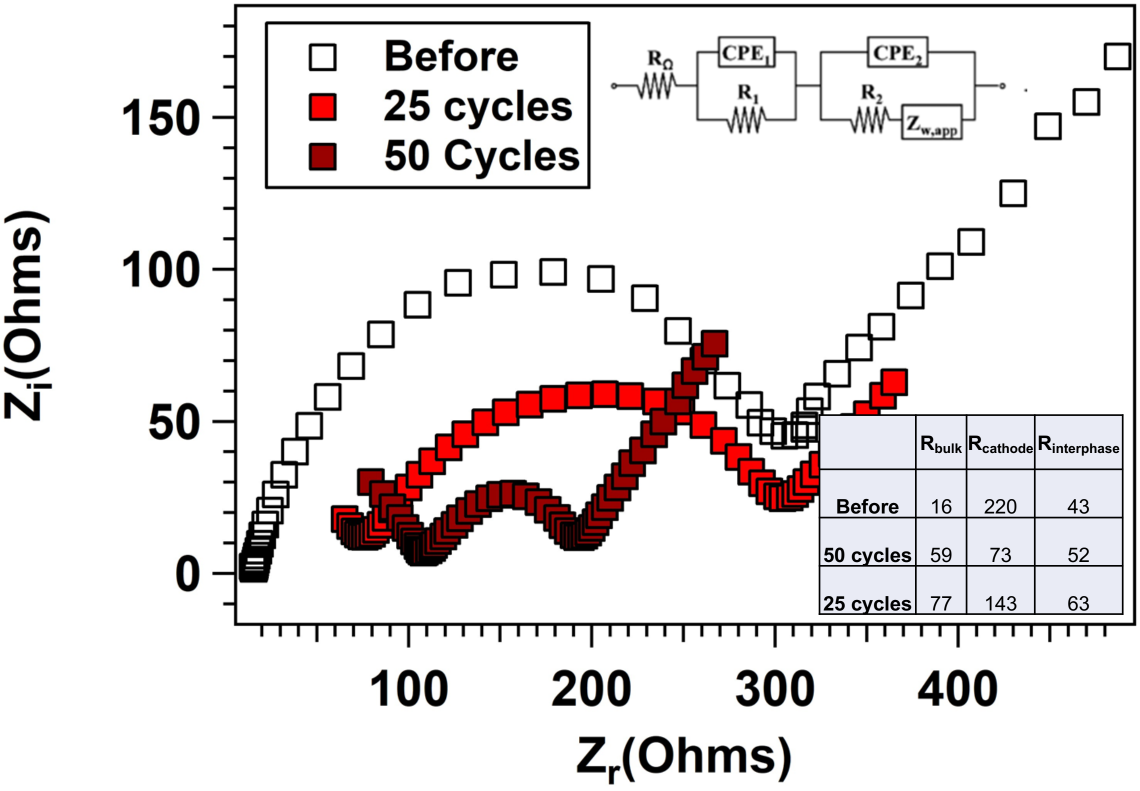

Supplementary Fig. 13: Impedance of Li@SPI|electrolyte|NMC full cell at different cycle numbers. The top inset shows the circuit model to extract the resistance values at the different interfaces and bulk electrolyte. The bottom right inset shows the resistance extracted from the impedance spectra using the same model

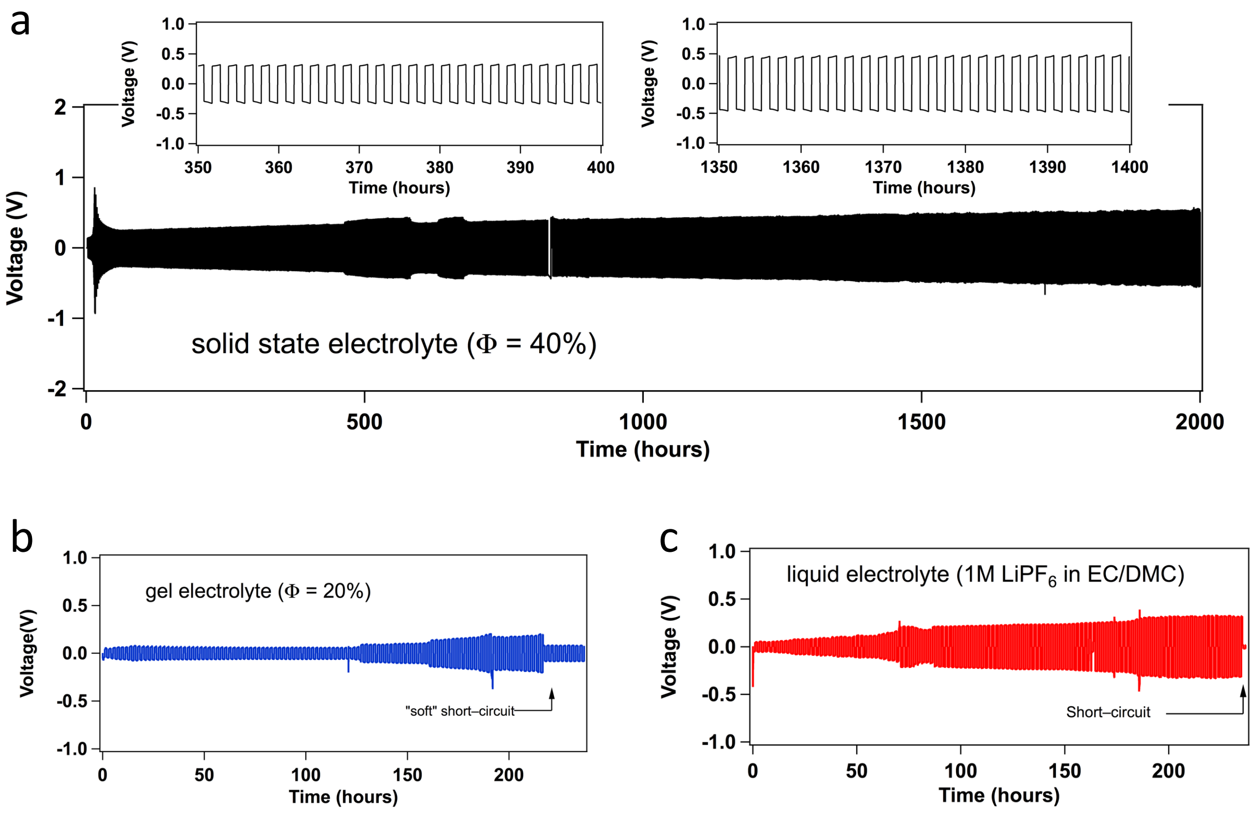

Supplementary Fig. 14: Comparison of symmetric cell cycling using different electrolyte mechanics: (a) Symmetric lithium metal battery cycling at a rate of 0.5mA/cm^2 with each half cycle being one hour. The electrolyte utilized was solid state polymer comprising of polymer network and diglyme, with PEGDMA content of 40% and with the salt $LiNO_3$ (Li:EO = 0.10). The thickness of the solid polymer electrolyte was ~400μm. The inset shows the expanded voltage profiles at different time of cycling **(b)** cycling using same conditions as a, however the electrolyte used with PEGDMA content of 20% that has gel-like texture; **(c)** results for liquid electrolyte of 1M $LiPF_6$ in EC/DMC (without any separator), instead an PTFE O-ring was used.

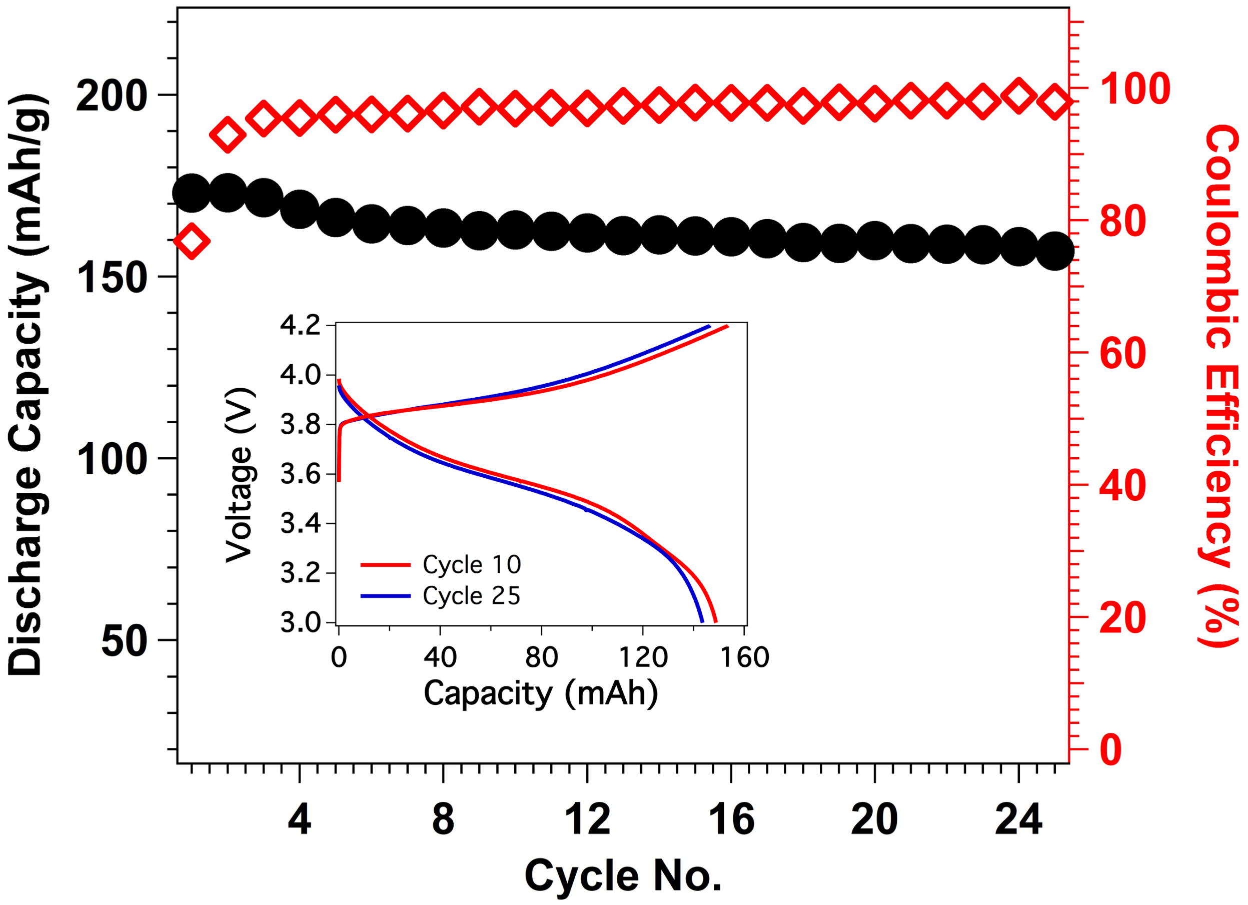

Supplementary Fig. 15: Cycling performances for Li||NMC cell operated at a C-rate of C/5. Here the electrolyte utilized was an all-solid state polymer electrolyte that comprises of the polymer network and diglyme, with PEGDMA content of 40% and with the salt $LiNO_3$ (Li:EO = 0.10) and 0.4M LiBOB as additive. The thickness of the solid polymer electrolyte was ~400μm. The capacity of cathode is $2mAh/cm^2$. The cathode surface was wetted by liquid electrolyte of diglyme-$LiNO_3$ (Li:EO = 0.1) and 0.4M LiBOB.
